# Non-Invasive Brain Stimulation Techniques and Effects on Neuromodulation and Athletic Performance in Combat Sports: A Systematic Review

**DOI:** 10.3390/brainsci16101052

**Published:** 2026-09-30

**Authors:** Rafael Pereira Azevedo Teixeira, Vanessa Teixeira Müller, Daniel Rodrigues Lopes, Marcson Love Maquiné de Freitas, Maycon da Cunha Herzog, Dany Alexis Sobarzo Soto, Ciro José Brito, Bianca Miarka

**Affiliations:** 1 Laboratory of Psychophysiology and Performance in Sports and Combats, Department of Physical Education, Federal University of Rio de Janeiro, Av. Carlos Chagas Filho, 540-Cidade Universitária, Rio de Janeiro 21941-599, RJ, Brazil; texera.123@gmail.com (R.P.A.T.); vanessatmuller@yahoo.com.br (V.T.M.); daniellopesjudo@hotmail.com (D.R.L.); mrcsnlove@gmail.com (M.L.M.d.F.); maycon.herzog@gmail.com (M.d.C.H.); 2 Magister en Ciencias la Actividad Física y Deportes Aplicadas al Entrenamiento Rehabilitación y Reintegro Deportivo, Universidad Santo Tomás, Puerto Montt 5500000, Chile; danysobarzo@santotomas.cl; 3 Programa de Pós-Graduação em Educação Física, Departamento de Educação Física, Universidade Federal de Viçosa, Viçosa 36570-900, Brazil

**Keywords:** neuromodulation, martial arts, athletic performance, cognitive performance, motor performance, reaction time, sports neuroscience

## Abstract

**Highlights:**

**What are the main findings?**

- Non-invasive brain stimulation (especially tDCS) showed potential to improve reaction time, selective attention, neuromuscular activation, and fatigue resistance in combat sport athletes.
- Despite promising findings, the certainty of evidence ranged from low to very low due to methodological limitations, small sample sizes, and heterogeneous stimulation protocols.

**What are the implications of the main findings?**

- NIBS, particularly tDCS, may have potential as a complementary strategy for modulating selected cognitive, motor, and psychophysiological outcomes in combat sports; however, the current low-to-very-low certainty of evidence does not support routine implementation.
- Further large-scale randomized controlled trials with standardized protocols are needed before routine practical implementation in athletic settings.

**Abstract:**

Background: Non-invasive brain stimulation (NIBS), including transcranial direct current stimulation (tDCS) and transcranial magnetic stimulation (TMS), has emerged as a potential ergogenic strategy for modulating neural activity and enhancing cognitive and physical performance in sports. However, evidence regarding its application in combat sports remains limited, heterogeneous, and fragmented. This study aimed to systematically synthesize the effects of NIBS on neuromodulation and cognitive, physical, and psychophysiological performance-related outcomes in combat sport athletes. Although NIBS encompasses several stimulation techniques, the evidence identified in combat sport athletes in this review predominantly involved tDCS. Methods: A systematic review was conducted in accordance with the PRISMA 2020 guidelines and registered in PROSPERO (CRD420251150915). Searches were performed in CAPES, PubMed, Scopus, and SPORTDiscus using predefined keywords related to combat sports and brain stimulation techniques. Studies were included if they involved combat sport athletes and investigated the effects of NIBS on performance-related outcomes. Data extraction included study design, sample characteristics, stimulation protocols, and main findings. Methodological quality was assessed using the Cochrane Risk of Bias 2 (RoB 2) tool for randomized studies and ROBINS-I for non-randomized studies, while certainty of evidence was evaluated using the GRADE framework. Results: Nine studies were included in the final synthesis (eight identified through database searching and one through other methods/manual reference screening), encompassing athletes from taekwondo, judo, boxing, mixed martial arts, fencing, and Brazilian jiu-jitsu. Most studies applied tDCS protocols targeting the primary motor cortex (M1) or the dorsolateral prefrontal cortex (DLPFC), with intensities ranging from 1 to 2 mA and stimulation durations between 10 and 20 min. tDCS demonstrated potential benefits in reaction time, selective attention, memory, and resistance to fatigue, as reflected by increased time to task failure. Additionally, enhancements in neuromuscular activation and reductions in anxiety were observed. However, results were heterogeneous, and some studies reported no significant effects on performance-related variables. Risk-of-bias assessment revealed methodological limitations across studies, and GRADE indicated low-to-very-low certainty of evidence for most outcomes. Conclusions: tDCS may influence selected cognitive, neuromuscular, and fatigue-related outcomes in combat sport athletes; however, the direction and magnitude of these effects are inconsistent across studies. Given the substantial methodological heterogeneity and the low-to-very-low certainty of evidence, no definitive conclusions regarding efficacy or routine practical application can currently be drawn. Further large-scale, standardized, high-quality randomized controlled trials are needed.

## 1. Introduction

Various brain stimulation techniques have been increasingly recognized as important tools in the field of neuromodulation, substantially contributing to the understanding of brain function and neurophysiological regulation [1,2,3,4,5,6,7,8]. In recent years, these techniques have attracted growing scientific interest not only as investigative tools in neuroscience but also as therapeutic and performance-enhancing interventions capable of modulating cortical excitability, synaptic plasticity, and functional connectivity across neural networks [2,6]. Such modulation may influence multiple domains relevant to human performance, including motor output, cognitive processing, fatigue perception, and sensorimotor integration. In this context, high-quality systematic reviews, particularly those conducted and reported according to standardized guidelines such as PRISMA (Preferred Reporting Items for Systematic Reviews and Meta-Analyses), occupy one of the highest levels in the hierarchy of scientific evidence, as they provide a critical, transparent, and reproducible synthesis of available findings, thereby reducing individual bias and strengthening the reliability of conclusions [9]. This approach is particularly relevant in review studies involving non-invasive brain stimulation (NIBS), such as transcranial direct current stimulation (tDCS) and transcranial magnetic stimulation (TMS), due to the substantial heterogeneity in stimulation parameters, cortical targets, electrode montages, intervention timing, and outcome measures reported across studies [10,11,12,13,14,15,16,17,18,19,20]. These techniques have demonstrated considerable potential both in the treatment of neurological and psychiatric disorders, such as social anxiety [1], and in the enhancement of cognitive and physical performance in healthy individuals and athletes [2].

Combat sport performance is determined by an integrated combination of physical, cognitive, technical, and tactical capacities, ranging from muscular strength and power to highly refined technical and tactical skills required for competitive success [7]. In addition to these physical demands, rapid decision-making, perceptual anticipation, inhibitory control, and emotional regulation are essential for performance in dynamic, high-pressure, and unpredictable combat environments [7]. Furthermore, each combat sport modality presents distinct physiological and neurocognitive demands. For instance, in Mixed Martial Arts (MMA), aerobic capacity, anaerobic power, and sport-specific physiological assessments, such as submaximal and supramaximal tests, are highly relevant determinants of athletic performance [8]. Therefore, performance-related outcomes may vary substantially depending on the specific combat sport and its underlying physiological and neurocognitive requirements. This variability suggests that neuromodulatory interventions may elicit sport-specific effects according to the neural networks targeted, particularly those associated with motor execution (e.g., primary motor cortex) and executive control (e.g., dorsolateral prefrontal cortex) [10,11,12,13,14,15,16,17,18,19].

However, analysis of the current literature reveals a lack of consensus regarding the effects of NIBS on performance in combat sports [10,11,12,13,14,15,16,17,18,19]. While some studies report significant improvements in variables such as reaction time, selective attention, muscle activation, fatigue resistance, and sport-specific performance, others report no significant effects or inconsistent findings [10,11,12,13,14,15,16,17,18,19]. This heterogeneity may be explained by substantial methodological differences, including variations in stimulation protocols (e.g., intensity, duration, stimulation timing, montage, and cortical target), sample characteristics (e.g., competitive level, sex, training background, and sport modality), and outcome measures [10,11,12,13,14,15,16,17,18,19]. Therefore, a critical synthesis of the available evidence is necessary to integrate these findings and provide a more robust understanding of the true magnitude, efficacy, and practical applicability of NIBS in combat sport performance.

Despite the growing scientific interest in the application of NIBS to enhance athletic performance, evidence related to combat sports remains limited, heterogeneous, and highly fragmented. To date, no systematic review has comprehensively synthesized the available evidence regarding the effects of NIBS in combat sport athletes, highlighting an important gap in the literature. Therefore, the present systematic review aims to critically analyze and synthesize the existing literature on the use of NIBS in combat sport athletes, focusing on its neuromodulatory effects and potential impact on performance.

It was hypothesized that NIBS may influence performance-related outcomes; however, the available evidence in combat sport athletes was expected to predominantly involve tDCS. By integrating the available findings, this review seeks to provide a more robust understanding of the efficacy, limitations, and practical applicability of these techniques for sports performance enhancement. Additionally, this review evaluates the methodological quality, risk of bias, and certainty of evidence of the included studies to strengthen the interpretation and clinical applicability of the available evidence.

## 2. Materials and Methods

### 2.1. Study Design

The present study was conducted in accordance with the Preferred Reporting Items for Systematic Reviews and Meta-Analyses 2020 (PRISMA 2020) guidelines [9], and the completed PRISMA 2020 checklist is provided as Supplementary Material (Table S1). The review protocol was registered in the International Prospective Register of Systematic Reviews (PROSPERO, https://www.crd.york.ac.uk/PROSPERO/view/CRD420251150915, accessed on 25 September 2026).

The review protocol was established a priori and followed rigorous methodological procedures to ensure transparency, reproducibility, and methodological rigor. Study selection, data extraction, risk-of-bias assessment, and certainty-of-evidence evaluation were performed according to the registered protocol, aiming to minimize potential sources of bias and strengthen the validity and reliability of the findings.

### 2.2. Search Strategy

The databases used for this review were CAPES, PubMed, Scopus, and SPORTDiscus. These databases were selected due to their broad coverage of biomedical, sports science, and interdisciplinary research relevant to neuromodulation and athletic performance. The literature search was conducted until January 2026 (final search performed on 30 January 2026), using a structured strategy based on a preliminary literature review, which allowed the identification of the main keywords relevant to the research topic. Boolean operators (AND, OR) were applied to combine terms related to combat sports and non-invasive brain stimulation techniques. The search strategies were adapted according to the specific requirements of each database. No restrictions were applied regarding publication date, and studies published in English were considered eligible. No additional filters regarding study design or participant characteristics were applied during the database search. Additionally, reference lists of included studies were manually screened to identify potentially relevant articles not captured in the database search. Manual screening of reference lists was performed to reduce the risk of missing eligible studies. The complete search strategy is presented in Table 1.

### 2.3. Inclusion and Exclusion Criteria

The eligibility criteria were defined a priori based on the PICOS framework (Population, Intervention, Comparator, Outcomes, and Study Design) and informed by prior literature to ensure a systematic and reproducible selection of studies.

Population (P): Studies involving recreational, competitive, or elite athletes engaged in combat sports (e.g., judo, boxing, taekwondo, mixed martial arts, Brazilian jiu-jitsu, fencing, and related disciplines) were considered eligible. Studies involving mixed samples were included only when combat sport athletes could be clearly identified and analyzed separately.

Intervention (I): Application of non-invasive brain stimulation techniques, including transcranial direct current stimulation, transcranial magnetic stimulation, and transcranial random noise stimulation.

Comparator (C): Studies with active versus sham stimulation, control groups, crossover comparisons, or within-subject pre–post comparisons were considered eligible.

Outcomes (O): Studies assessing performance-related outcomes, including physical (e.g., strength, endurance, reaction time), cognitive (e.g., attention, memory, executive function), or psychophysiological variables relevant to sports performance.

Study Design (S): Randomized controlled trials, non-randomized controlled studies, quasi-experimental studies, and experimental studies with acute, crossover, or repeated interventions were included.

Studies were excluded if they involved participants not engaged in combat sports, included clinical populations, or focused exclusively on clinical or therapeutic outcomes without relevance to sports performance or neuromodulation. Additionally, reviews (systematic or narrative), meta-analyses, editorials, letters to the editor, conference abstracts without full text, and case reports were excluded. Only studies published in English and available in full text were considered eligible.

### 2.4. Study Selection Process

**Figure 1:**
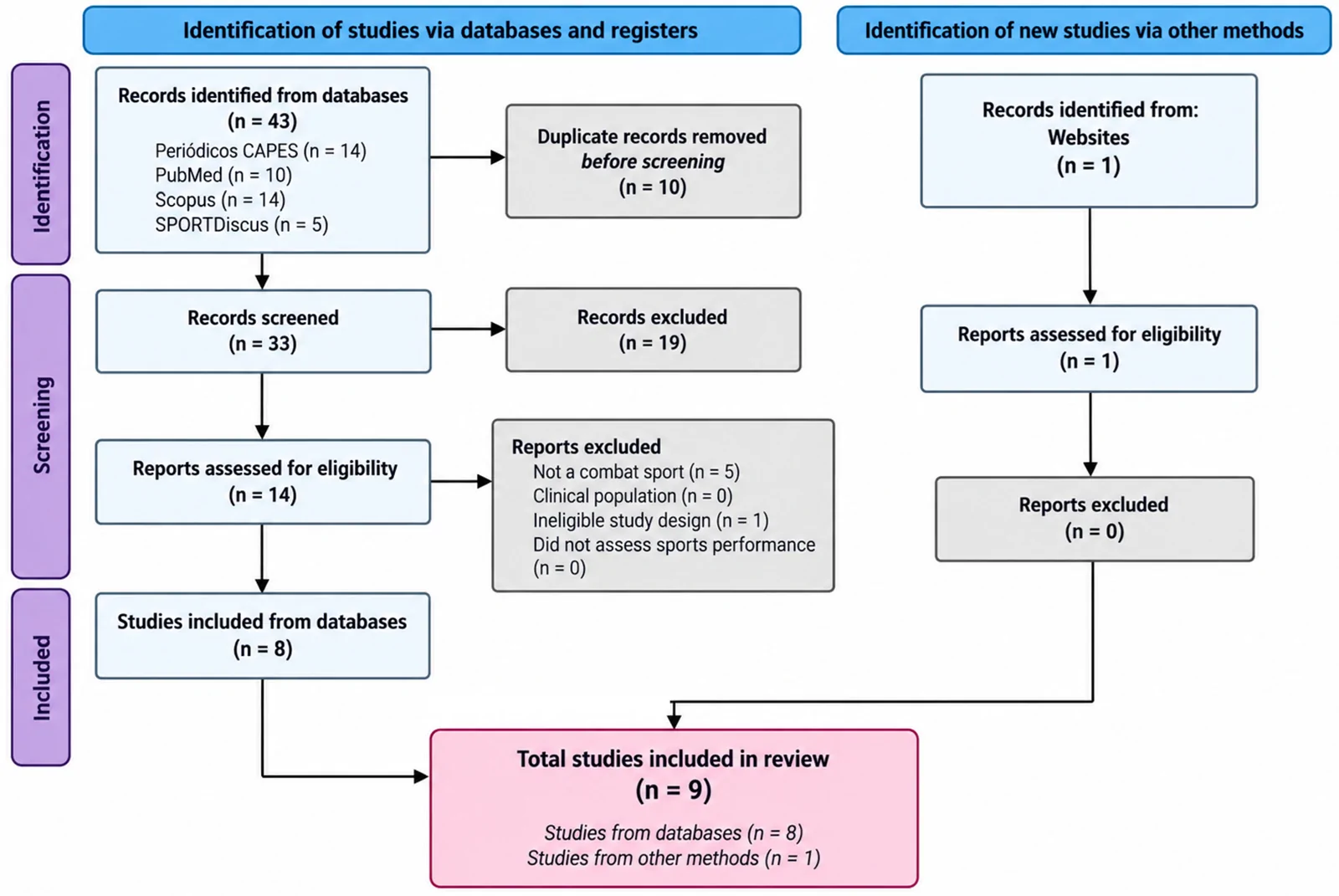
Flow diagram of the identification, screening, eligibility, and inclusion of studies according to PRISMA.

After applying the search strategies, the Rayyan platform was used to manage, organize, and screen the identified records in a systematic and blinded manner, in accordance with the PRISMA 2020 guidelines (Figure 1).

Duplicate records were initially identified and removed using the platform’s automatic detection tool, followed by manual verification to ensure accuracy. Subsequently, two independent reviewers performed the screening process to reduce selection bias and improve the reliability of study selection.

The screening process was conducted in three sequential stages: (1) title screening, (2) abstract screening, and (3) full-text assessment, based on the predefined inclusion and exclusion criteria. Inter-reviewer disagreements were resolved through discussion, and when consensus could not be reached, a third reviewer was consulted for final decision-making.

The study selection process, including the number of records identified, screened, excluded (with reasons), and included in the final synthesis, is presented in the PRISMA 2020 flow diagram (Figure 1).

### 2.5. Risk-of-Bias Assessment

The methodological quality and risk of bias of the included studies were assessed independently by two reviewers according to study design. For randomized controlled trials and randomized crossover studies, the Cochrane Risk of Bias 2 (RoB 2) tool was used, following the recommendations of Sterne et al. [20]. The RoB 2 tool evaluates five domains: (1) bias arising from the randomization process, (2) bias due to deviations from intended interventions, (3) bias due to missing outcome data, (4) bias in measurement of the outcome, and (5) bias in selection of the reported result.

For non-randomized and quasi-experimental studies, the Risk Of Bias In Non-randomized Studies of Interventions (ROBINS-I) tool was applied, following the indications of Sterne et al. [21]. This instrument assesses seven domains: (1) bias due to confounding, (2) bias in selection of participants, (3) bias in classification of interventions, (4) bias due to deviations from intended interventions, (5) bias due to missing data, (6) bias in measurement of outcomes, and (7) bias in selection of the reported result. Studies were classified as presenting low, moderate, serious, or critical risk of bias.

The risk-of-bias assessments were performed independently by two reviewers. Any disagreements in domain-level judgments were resolved through discussion and consensus between the two reviewers. No disagreements required adjudication by a third reviewer. Reviewer-specific assessments were not systematically retained for reporting purposes; therefore, quantitative inter-rater agreement could not be retrospectively calculated. The potential direction of bias was not formally assessed; therefore, only domain-level and overall risk-of-bias judgments are reported. The results of the risk-of-bias assessment were summarized in results.

### 2.6. Certainty of Evidence Assessment (GRADE)

The certainty of evidence for the main outcomes included in this review was assessed using the Grading of Recommendations Assessment, Development and Evaluation (GRADE) framework. The quality of evidence was evaluated across five domains: risk of bias, inconsistency, indirectness, imprecision, and publication bias. Evidence derived from randomized controlled trials was initially classified as high certainty, whereas evidence from non-randomized or quasi-experimental studies was initially classified as low certainty. The certainty level was subsequently downgraded or upgraded according to the presence of methodological limitations, heterogeneity among findings, indirectness of the evidence relative to the review question, imprecision related to sample size or confidence in estimates, and potential publication bias. The overall certainty of evidence for each major outcome was categorized as high, moderate, low, or very low. Given the heterogeneity of stimulation protocols, participant characteristics, and outcome measures, the GRADE assessment was applied qualitatively to the principal outcome domains, including reaction time, cognitive performance, neuromuscular activation, anxiety, and fatigue-related outcomes.

### 2.7. Data Extraction

A standardized data extraction table was developed to summarize the main characteristics of the included studies and support the synthesis of the review. Data extraction was performed independently by two reviewers using a predefined extraction form to minimize bias and improve data reliability. Any disagreements were resolved through discussion, and when necessary, a third reviewer was consulted to achieve consensus.

A meta-analysis was not performed due to substantial heterogeneity across three dimensions: interventions (stimulation modality, cortical target, intensity, and session parameters varied widely across studies), outcomes (different types of instruments were used across cognitive, neuromuscular, and psychophysiological domains, precluding a common effect metric), and study design (randomized, crossover, and also some experimental studies were combined, several with small samples). Pooling under these conditions would have produced a statistically computable but clinically uninterpretable summary effect.

The table included the following information: author and year of publication, study objective, study design and sample characteristics, sport modality, instruments used, type of stimulation, stimulation parameters (e.g., intensity, duration, and cortical target), comparator condition, and main findings. Whenever available, methodological quality indicators and relevant statistical outcomes were also recorded.

## 3. Results

Our results demonstrated that, after applying the eligibility criteria, a total of nine studies were included in the final synthesis: eight were identified through database searching and one additional study was identified through other methods/manual reference screening, as shown in the PRISMA flow diagram. The studies covered modalities such as taekwondo, boxing, mixed martial arts, fencing, and Brazilian jiu-jitsu and were systematically characterized according to sample, sport modality, and methodological features. Their detailed description and categorization are presented in Table 2.

**Figure 2:**
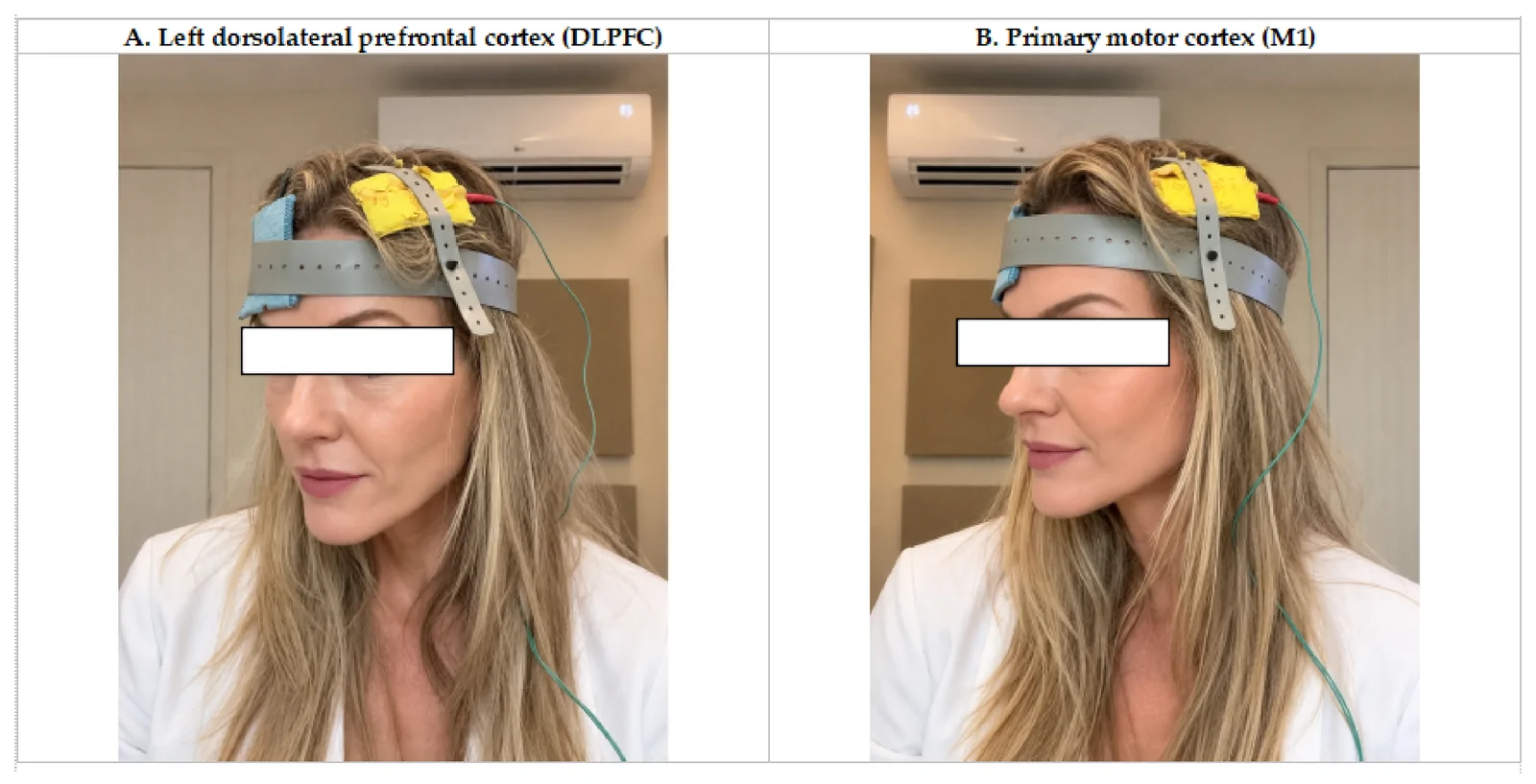
Representative tDCS electrode placements used to illustrate the two principal cortical targets identified in the included studies: (**A**) left dorsolateral prefrontal cortex (DLPFC) and (**B**) primary motor cortex (M1). Photographs are illustrative of electrode positioning and do not represent neuronavigated localization.

tDCS was the most commonly used technique as illustrated in Figure 2, with protocols ranging from 1 to 2 mA and durations between 10 and 20 min (Table 3), predominantly targeting the primary motor cortex and prefrontal regions, areas recognized for their role in motor control and decision-making [4].

Most studies employed controlled experimental designs, including sham and experimental conditions, with a significance level set at *p* < 0.05, as shown in Table 4 and Table 5, categorized according to DLPFC and M1 stimulation, respectively.

The main findings indicated that active stimulation was associated with significant reductions in reaction time and improvements in selective attention [10], suggesting enhanced sensorimotor processing speed and more efficient perception–action coupling. In terms of physical outcomes, some studies reported improvements in muscle activation, particularly in the gluteus medius and maximus, semitendinosus, biceps femoris, and gastrocnemius muscles [11]. Regarding strength in elite male Brazilian Jiu-Jitsu (BJJ) athletes, results showed an increase in time to failure during an isometric handgrip task performed at 60% of maximal voluntary contraction (MVC), with higher mean values observed in the anodal tDCS (a-tDCS) condition compared to sham [18].

In addition, the application of tDCS during the pre-competitive period was associated with improvements in psychophysiological indicators, including reduced anxiety and enhanced technical and tactical readiness, as well as increased effectiveness in offensive, defensive, and technical execution actions [14]. The same study also demonstrated significant improvements in cognitive domains, including attention, memory, and information processing, accompanied by reduced stress following active stimulation [14]. Furthermore, tDCS application in elite fencers resulted in a significant reduction in injury-related anxiety and improved sports performance [16].

**Figure 3:**
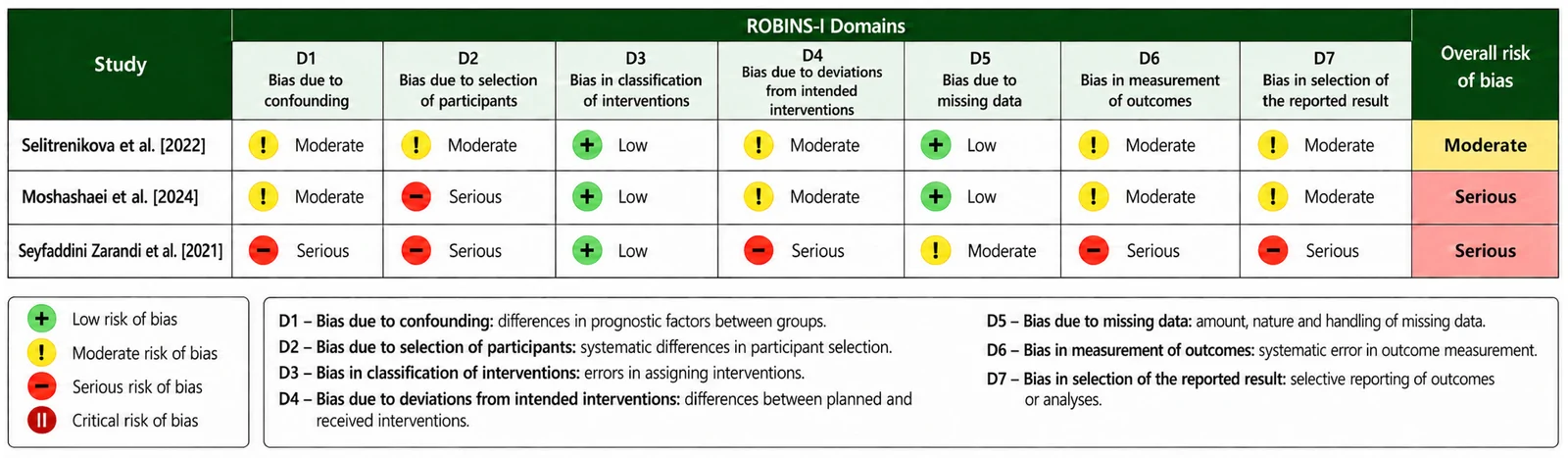
Risk-of-bias assessment of randomized studies using RoB 2 [14,15,16].

The methodological quality of the included studies varied according to study design. Randomized trials were evaluated using the RoB 2 tool, with most studies classified as presenting low risk or some concerns in specific domains (Figure 3).

Overall, the RoB 2 assessment indicated predominantly low risk of bias or some concerns across the randomized studies, with no studies classified as having an overall high risk of bias. The main concerns were related to the randomization process and selection of the reported results.

**Figure 4:**
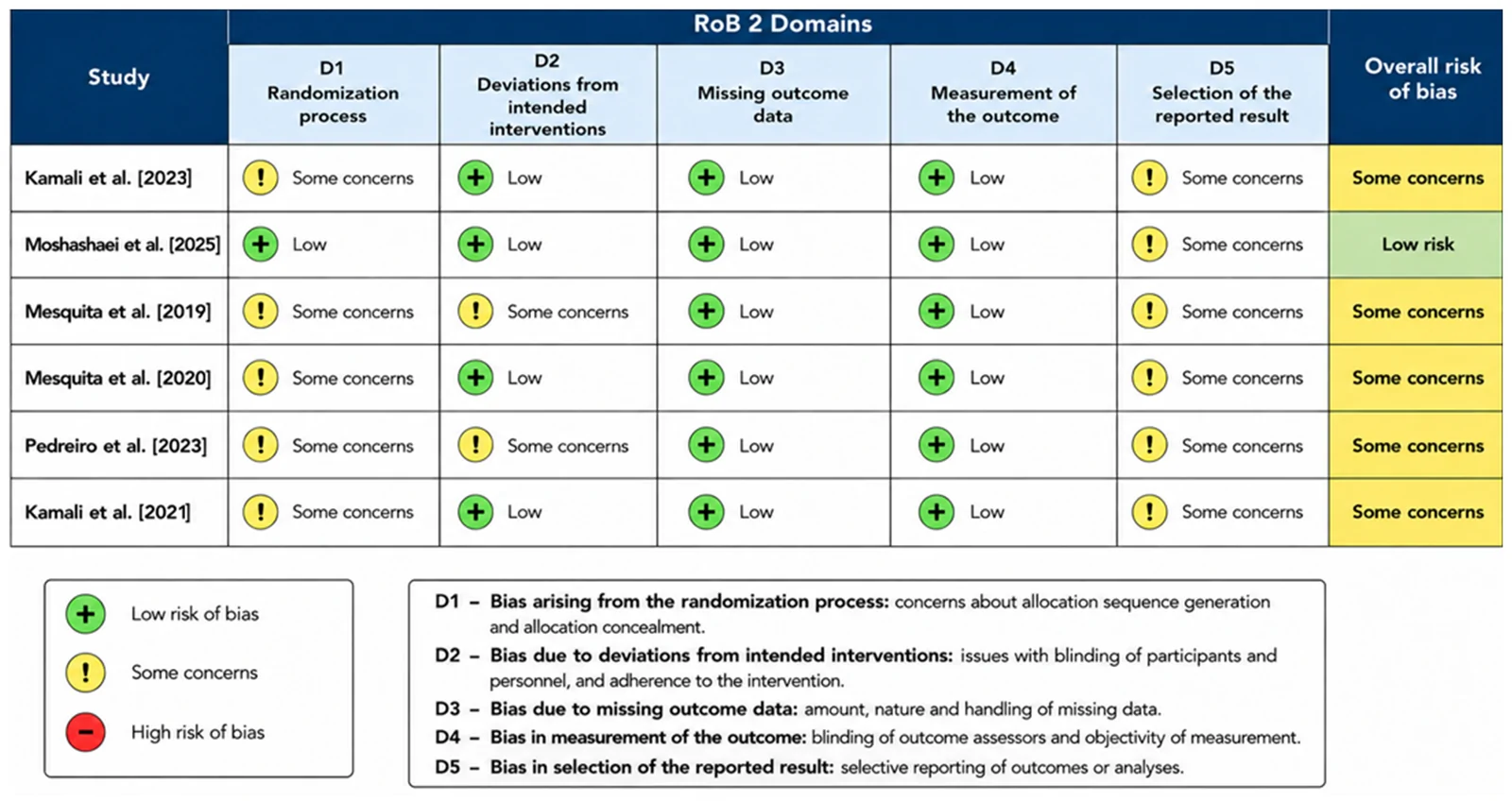
Risk-of-bias assessment of non-randomized studies using ROBINS-I [10,11,12,17,18,19].

Non-randomized and quasi-experimental studies were assessed using ROBINS-I, showing moderate to serious risk of bias, particularly in domains related to confounding and participant selection (Figure 4).

The certainty of evidence for the main outcome domains was qualitatively assessed using the GRADE (Grading of Recommendations Assessment, Development and Evaluation) framework (Table 6).

Table 6 demonstrates that the certainty of evidence ranged from very low to low, primarily due to methodological limitations, small sample sizes, heterogeneity in stimulation protocols, and variability in outcome measures across studies. Evidence related to reaction time was classified as low certainty, despite consistent findings suggesting that performance may improve following active stimulation.

This classification was mainly due to small sample sizes, heterogeneous stimulation parameters, and limitations in randomization and blinding procedures among included studies.

Evidence regarding cognitive outcomes, including selective attention, memory, and executive function, was rated as low-to-very-low certainty. Although some studies reported improvements after stimulation, substantial heterogeneity was observed in cognitive assessment tools, cortical targets, and participant characteristics [10,11,12,13,14,15,16,17,18,19]. Additionally, indirectness of evidence was noted in studies involving populations with characteristics not fully representative of competitive combat sport athletes.

Evidence for neuromuscular activation was classified as low certainty. Positive effects were observed in lower-limb muscle activation following tDCS; however, conclusions were limited by small sample sizes, protocol variability, and restricted external validity due to sport-specific samples.

Evidence related to anxiety and psychophysiological outcomes was rated as very low certainty, as conclusions were based on a limited number of studies and subjective outcome measures, increasing susceptibility to performance and detection bias.

Finally, evidence for fatigue-related outcomes, including time to task failure and endurance performance, was classified as low certainty. Although some findings suggested improvements in fatigue resistance following stimulation, inconsistency across study designs and intervention protocols reduced confidence in the estimates.

## 4. Discussion

Findings from this systematic review suggest that tDCS may exert beneficial effects on physical, cognitive, and psychophysiological aspects of performance in combat sport athletes, although these effects should be interpreted cautiously due to the overall low certainty of evidence. These findings are consistent with recent meta-analytic evidence indicating that tDCS effects on sport-specific performance are highly heterogeneous and protocol-dependent, with stimulation parameters and target region acting as significant moderators of outcome [22,23,24,25]. However, one meta-analysis reported a moderate overall effect with the most consistent results for M1-targeted protocols [23], while others have characterized the evidence as inconclusive or negligible-to-small once heterogeneity is accounted for [24,25], underscoring that any performance benefit of tDCS is likely conditional on specific dose and montage parameters rather than a general effect of the technique, a pattern that was also reflected in the wider variability of findings reported across these meta-analyses. Meta-analytic evidence has shown that tDCS may produce small but statistically significant improvements in motor and cognitive performance tasks, as well as modest enhancements in professional athletes, particularly in endurance and competitive or cooperative sport-specific skills [23]. However, the risk-of-bias assessments presented in Figure 2 (Risk-of-bias assessment of randomized studies using RoB 2) [20] and Figure 3 (Risk-of-bias assessment of non-randomized studies using ROBINS-I) [21] revealed important methodological limitations across the included studies. These limitations, particularly those related to allocation concealment, confounding, selective reporting, and incomplete blinding procedures, contributed substantially to downgrading evidence certainty in the GRADE assessment. In addition to methodological limitations, evidence certainty was further reduced due to inconsistency across stimulation protocols, imprecision resulting from small sample sizes, indirectness of certain study populations, and possible publication bias favoring positive ergogenic findings. Consequently, according to the GRADE assessment, the certainty of evidence ranged from low to very low for most outcomes, limiting confidence in the magnitude and reproducibility of the reported effects.

From a neurophysiological perspective, the effects of NIBS likely extend beyond simple modulation of cortical excitability and may involve broader neural systems associated with performance regulation [23,24,25,26,27]. NIBS has been proposed to influence dopaminergic pathways, potentially modulating motivation, reward processing, effort perception, and decision-making under pressure, all of which are highly relevant during high-intensity competitive tasks [24,28]. Additionally, alterations in cortical oscillatory activity, particularly within alpha and beta frequency bands, may contribute to improvements in motor planning, attentional allocation, inhibitory control, and sensorimotor integration [29,30]. These mechanisms are especially relevant in combat sports, where successful performance depends on rapid visuomotor processing, perception–action coupling, tactical flexibility, and precise motor execution under temporal pressure.

The potential influence of NIBS on central fatigue mechanisms further supports its possible role in sustaining performance under physically demanding conditions. Specifically, modulation of motor cortex inhibition and corticospinal excitability may alter motor unit recruitment and reduce perceived exertion during repeated high-intensity actions [26,27,28,29]. For example, evidence from Kamali et al. [10] demonstrated significant reductions in reaction time and improvements in selective attention following stimulation, supporting the hypothesis that tDCS may enhance sensorimotor processing speed and perception–action coupling. Similarly, Moshashaei et al. [11] reported increased activation of lower limb muscles, suggesting enhanced corticospinal excitability and improved neuromuscular efficiency. Regarding endurance-related outcomes, Pedreiro et al. [18] observed increased time to task failure during isometric contraction, suggesting reduced central fatigue and greater capacity to sustain submaximal force production. Beyond motor performance, Seyfaddini Zarandi et al. [16] demonstrated reductions in anxiety and improvements in performance, highlighting the possible influence of prefrontal modulation on emotional regulation and decision-making under competitive stress. Furthermore, cognitive improvements reported in a recent study on Tai chi by Xu et al. [13], including enhanced attention, memory, and executive function, reinforce the multidimensional effects of neuromodulation on performance-relevant domains. However, interpretation of Xu et al. [13] should remain cautious due to the indirectness of the study population relative to competitive combat sport athletes, and this limitation directly contributed to downgrading certainty of evidence for indirectness in the GRADE assessment. In contrast, findings from Mesquita et al. [12], which reported no significant improvements in sport-specific performance (countermovement jump height, total kicks, and kick decrement index; all *p* > 0.05), differed from studies reporting significant effects in at least three identifiable ways. First, the stimulation dose was lower: a bilateral M1 montage (C3–C4) delivered at 1.5 mA for 15 min (cumulative charge ≈ 22.5 mA·min) was used, compared with the unilateral extracephalic M1 montage at 2 mA for 20 min (≈40 mA·min) used by Moshashaei et al. [15], which did report significant increases in muscle activation. Second, the bilateral bihemispheric montage itself may generate opposing current flow patterns across hemispheres, potentially attenuating the net change in corticospinal excitability relative to unilateral montages with an extracephalic reference, which maximize unidirectional current flow through the target region. Third, the outcome measures differed in nature: Mesquita et al. [12] assessed integrated, whole-body sport-specific performance (jump height, repeated kicking output), which depends on technique, pacing, and peripheral muscular fatigue in addition to central neural drive, whereas studies reporting significant effects predominantly used more proximal, isolated neurophysiological outcomes (e.g., single-muscle EMG amplitude, reaction time). Complex performance outcomes of this kind are inherently more variable and less directly coupled to cortical excitability changes, which may explain the null result despite a plausible mechanism of action.

Classical TMS studies have demonstrated that protocols such as iTBS can induce sustained changes in cortical excitability, thereby influencing strength production, motor output, and power generation [4,5]. Although most studies included in this review investigated tDCS, the physiological mechanisms underlying these interventions appear interconnected, involving modulation of neural activation during high-intensity motor tasks. In combat sports, where milliseconds may determine offensive or defensive success, even small improvements in reaction time or motor planning may provide meaningful competitive advantages. Experimental evidence suggests that anodal tDCS may improve feedforward activation of lower limb musculature in taekwondo athletes, contributing to improved joint stability and motor efficiency [11]. Likewise, improvements in time to exhaustion during isometric handgrip tasks observed in elite Brazilian Jiu-Jitsu athletes suggest reduced fatigue and improved capacity to maintain submaximal force [18]. Narrative and systematic reviews further suggest that stimulation of cortical regions such as the primary motor cortex and dorsolateral prefrontal cortex may enhance communication between sensory, motor, and executive networks, facilitating faster and more accurate motor responses [22,24]. Collectively, these findings suggest a plausible hypothesis that neuromodulation may act through integrated motor and prefrontal circuits; however, the heterogeneous and low-certainty evidence does not establish consistent enhancement of motor efficiency, cognitive control, or emotional regulation across combat sport contexts.

Regarding performance, findings demonstrating increased muscle activation, improved fatigue resistance, reduced anxiety, enhanced technical and tactical readiness, and better memory and information processing are broadly consistent with meta-analyses reporting small positive effects of tDCS on strength, endurance, and visuomotor skills [25,26]. These findings indicate that tDCS may influence selected performance-related outcomes under specific protocols, but the available evidence is insufficient to characterize it as a generally effective ergogenic aid. This possibility may be particularly relevant in combat sports, where both physical and mental fatigue can compromise technical execution, force production, tactical decision-making, and reaction speed during a match [27]. Nevertheless, the included studies varied considerably in stimulation dose (intensity ranging from 1 to 2 mA and duration from 10 to 20 min per session, representing up to a two-fold difference in cumulative charge), electrode montage (unilateral vs. bilateral, cephalic vs. extracephalic reference), and stimulation schedule (single session vs. six sessions over two weeks), as highlighted in Table 2 and Table 3. Outcome measures were similarly heterogeneous, including isolated neurophysiological metrics (single-muscle EMG amplitude, simple reaction time), standardized cognitive scores (MoCA, Memory Quotient), and integrated sport-specific performance measures (countermovement jump, repeated kicking output, time to exhaustion) (Table 5). Each of the nine included studies employed a distinct combination of electrode montage, stimulation dose, and outcome domain that can be checked in Table 2, Table 3 and Table 5, which restricts direct comparison across studies and prevents definitive recommendations regarding optimal intervention parameters.

This review has several limitations that should be considered when interpreting the findings. First, the number of included studies was relatively small, and most investigations involved limited sample sizes, reducing statistical power and limiting generalizability. Second, substantial heterogeneity was observed across studies regarding stimulation parameters, targeted brain regions, intervention timing, outcome measures, and athlete characteristics. This variability precludes definitive conclusions regarding optimal stimulation protocols. Third, the predominance of short-term experimental designs restricts understanding of long-term effects and the persistence of neuromodulatory adaptations. Additionally, although several studies employed randomized or controlled designs, inconsistencies were observed in blinding procedures, allocation concealment, and the use of appropriate sham conditions. These issues increase susceptibility to placebo effects and performance bias, particularly in studies assessing subjective or effort-dependent outcomes. Expectancy effects should also be considered, as participants may perceive sensory differences between active and sham stimulation, potentially compromising blinding integrity. Finally, potential publication bias cannot be excluded, as studies reporting positive ergogenic effects may be more likely to be published than studies with null findings.

In high-performance sports, marginal gains may determine the difference between victory and defeat, particularly in combat sports, where outcomes are frequently decided by fine temporal, technical, and tactical margins. Consequently, even modest improvements in reaction time, endurance, decision-making, or emotional control may translate into meaningful competitive advantages when applied in appropriate contexts [18]. However, one of the most important limitations in the current literature is the lack of clarity regarding dose–response relationships. Variations in stimulation intensity, duration, electrode montage, cortical target, and timing relative to exercise make it difficult to determine optimal stimulation parameters for performance enhancement. The absence of standardized protocols represents a major barrier to practical implementation and highlights the need for systematic investigations exploring parameter-specific effects [15,16,17,18,19,20,21,22,23,24,25,26,27,28,29,30].

The relevance of NIBS may be particularly pronounced in combat sports due to their unique performance demands. Combat disciplines require continuous perception–action coupling, rapid decision-making under uncertainty, motor inhibition, and emotional regulation in high-pressure environments. These characteristics suggest that neuromodulatory interventions targeting both motor and prefrontal regions may have amplified relevance in this context because they simultaneously influence physical execution and cognitive control processes essential for competitive success [18,28]. Application during pre-competition preparation or training sessions may help optimize reaction time, fatigue resistance, and cognitive readiness.

Nevertheless, safety findings obtained from general healthy and clinical populations should not be directly extrapolated to combat sport athletes. These athletes may be exposed to sport-specific conditions, including recent head impacts or concussion, rapid weight loss and dehydration, sleep restriction, acute fatigue, intensive training loads, and the use of medications or supplements, which could potentially influence individual responses to neuromodulation. Therefore, future studies should incorporate systematic pre-stimulation screening and prospective adverse-event monitoring. Headache, scalp discomfort, tingling, itching, burning sensations, dizziness, fatigue, skin reactions, mood changes, and neurological symptoms should be systematically recorded following stimulation sessions. Studies should also establish predefined stopping criteria and procedures for managing unexpected or serious adverse events. For TMS-based protocols, additional considerations should include seizure-risk screening, stimulation frequency and intensity, medications affecting seizure threshold, sleep deprivation, hearing protection, and appropriate operator training [31,32].

Future research should therefore address both the methodological and safety limitations identified in the current literature. Larger, adequately powered randomized controlled trials appear warranted; however, these studies should be conducted under controlled research conditions using standardized stimulation protocols, established safety parameters, appropriate participant screening, systematic adverse-event monitoring, predefined stopping criteria, and trained professional supervision. Future studies should also investigate long-term effects, sex-specific responses, dose–response relationships, blinding integrity, and interactions between neuromodulation and training adaptations. Multicenter trials using standardized outcome measures would further improve statistical power, reproducibility, and generalizability. Studies conducted in ecologically valid training and competitive settings are also needed to determine whether the observed effects translate into meaningful improvements in combat sport performance. Until such evidence is available, tDCS and other NIBS modalities should be considered experimental approaches rather than routinely recommended ergogenic strategies for combat sport athletes.

## 5. Conclusions

This review identified nine studies investigating non-invasive brain stimulation in combat sport athletes, with the available evidence predominantly involving tDCS. Eight studies were identified through database searching and one additional study through other methods/manual reference screening. Across these studies, effects on cognitive, neuromuscular, psychophysiological, and performance-related outcomes were heterogeneous.

Some studies reported improvements in reaction time, selective attention, specific measures of neuromuscular activation, anxiety, and fatigue resistance, whereas other outcomes showed no significant effects, including countermovement jump performance, repeated kicking measures, time to exhaustion, visuospatial memory, and handgrip strength. Accordingly, the findings should be considered preliminary and outcome- and protocol-dependent. Given the low-to-very-low certainty of evidence, the current literature does not support routine use of tDCS in combat sports, and its application should remain within controlled research settings.

Progress in this field will depend on methodological standardization in future trials, including consensus stimulation protocols reported according to prespecified cortical targets; randomized, double-blind, sham-controlled designs with formally verified blinding; adequately powered multicenter samples; a harmonized core outcome set; stratification by competitive level; and sport-specific, multi-session assessments rather than reliance on single-session laboratory tasks.

Given the differing physiological and neurocognitive demands across combat disciplines, future large trials should focus on a single modality or on clearly defined categories, such as striking-dominant sports (e.g., boxing, taekwondo, Muay Thai, MMA, and karate), in which reaction time and fatigue resistance during repeated high-intensity actions may be particularly relevant, and grappling-dominant sports (e.g., judo, Brazilian jiu-jitsu, and wrestling), in which isometric strength endurance and sustained neuromuscular output may be more central.

This approach would help determine whether NIBS effects are modality-specific or generalizable across groups of combat sports, rather than assuming a uniform ergogenic mechanism across all disciplines.

## Figures and Tables

**Table 1:** Detailed search strategies and Boolean operators applied across databases.

Database	Search Strategy
CAPES	(“martial art” OR “combat sport” OR “judo” OR “jiu jitsu” OR “Brazilian jiu jitsu” OR “boxing” OR “karate” OR “taekwondo” OR “muay thai” OR “kickboxing” OR “fencing” OR “capoeira” OR “sambo” OR “kung fu” OR “MMA” OR “wrestling”) AND (“tDCS” OR “tRNS” OR “dTMS” OR “tPCS” OR “HD-tDCS”)
PubMed	((“martial arts”[MeSH] OR “martial art” OR “judo” OR “jiu jitsu” OR “Brazilian jiu jitsu” OR “boxing” OR “karate” OR “taekwondo” OR “muay thai” OR “kickboxing” OR “fencing” OR “capoeira” OR “sambo” OR “kung fu” OR “combat sport” OR “MMA” OR “wrestling”)) AND ((“tDCS”[MeSH] OR “tDCS” OR “tRNS” OR “dTMS” OR “tPCS” OR “HD-tDCS”))
Scopus	TITLE-ABS-KEY (“martial art” OR “combat sport” OR “judo” OR “jiu jitsu” OR “Brazilian jiu jitsu” OR “boxing” OR “karate” OR “taekwondo” OR “muay thai” OR “kickboxing” OR “fencing” OR “capoeira” OR “sambo” OR “kung fu” OR “MMA” OR “wrestling”) AND TITLE-ABS-KEY (“tDCS” OR “tRNS” OR “dTMS” OR “tPCS” OR “HD-tDCS”)
SPORTDiscus	(“martial art” OR “combat sport” OR “judo” OR “jiu jitsu” OR “Brazilian jiu jitsu” OR “boxing” OR “karate” OR “taekwondo” OR “muay thai” OR “kickboxing” OR “fencing” OR “capoeira” OR “sambo” OR “kung fu” OR “MMA” OR “wrestling”) AND (“tDCS” OR “tRNS” OR “dTMS” OR “tPCS” OR “HD-tDCS”)

**Table 2:** Characteristics of participants included in the studies (combat sports athletes).

Study	Combat Sport	N	Age (Years)	Weight (kg)	Height (cm)	Experience (Years)
Kamali et al. [10]	Taekwondo	15	23.5 ± 3	67.4 ± 19	180 ± 7	5.2 ± 2.1
Moshashaei et al. [11]	Taekwondo	34	21.52 ± 5.78	60.12 ± 8.89	164 ± 5	5.68 ± 1.90
Mesquita et al. [12]	Taekwondo	19	19 ± 3	60.7 ± 6.9	171.7 ± 6.9	8.9 ± 5.0
Selitrenikova et al. [14]	MMA	20	22.5 ± 0.8	NI	NI	NI
Moshashaei et al. [15]	Taekwondo	34	21.96 ± 5.33	59.15 ± 9.20	163 ± 5	≥3
Seyfaddini Zarandi et al. [16]	Fencing	36	18.40 ± 2.51	NI	NI	NI
Mesquita et al. [17]	Taekwondo	12	19 ± 3	61.2 ± 8.5	172.1 ± 7.3	8.6 ± 5.6
Pedreiro et al. [18]	Brazilian Jiu-Jitsu	10	25.5 ± 5.7	77.1 ± 8.6	175 ± 6.0	>3
Kamali et al. [19]	Boxing	14	22.3 ± 3	75 ± 19	176 ± 16	3.2 ± 1.1

**Table 3:** Characteristics of tDCS protocols, including stimulated area and session parameters.

Study	Stimulated Area	Session Parameters
Kamali et al. [10]	Primary motor cortex (M1) and lumbar spinal segment (T12–L2)	2 sessions (72 h apart)
Moshashaei et al. [11]	M1 region	20 min at 2 mA
Mesquita et al. [12]	Bilateral M1 (C3 and C4, 10–20 EEG system)	1.5 mA for 15 min (rubber electrodes)
Selitrenikova et al. [14]	Anode: anterior prefrontal cortex; Cathode: shoulder	1–2 mA for 30 min
Moshashaei et al. [15]	Anode: M1 region; Cathode: left shoulder (extracephalic)	2 mA for 20 min
Seyfaddini Zarandi et al. [16]	DLPFC (F3 left hemisphere; F4 right hemisphere)	2 mA for 20 min per session (6 sessions over 2 weeks)
Mesquita et al. [17]	M1 region	1.5 mA for 15 min (extracephalic bihemispheric montage)
Pedreiro et al. [18]	DLPFC	2 mA for 20 min
Kamali et al. [19]	M1 region	0 to 2 mA for 13 min

**Table 4:** Effects of tDCS on cognitive outcomes (DLPFC-targeted studies).

Study	Variable	Group	Before	After	*p*-Value
Selitrenikova et al. [14]	Attention distribution	Control	13.5 ± 0.2	13.5 ± 0.2	<0.05
	Attention distribution	Active tDCS	13.2 ± 0.1	17.3 ± 0.2	
	Memory for numbers	Control	8.3 ± 0.4	8.3 ± 0.4	<0.05
	Memory for numbers	Active tDCS	8.4 ± 0.4	11.1 ± 0.3	
Seyfaddini Zarandi et al. [16]	Anxiety	Control	84.70 ± 19.42	84.70 ± 19.62	<0.05
	Anxiety	tDCS	70.92 ± 12.82	62.15 ± 12.28	
	Performance	Control	14.50 ± 2.24	15.07 ± 0.80	<0.05
	Performance	tDCS	12.05 ± 1.21	15.76 ± 1.09	
Pedreiro et al. [18]	Rating of perceived exertion	tDCS	–	7.5 ± 0.8	0.10
	Rating of perceived exertion	Sham	–	7.9 ± 0.4	
	Time to task failure (s)	tDCS	–	79.6 ± 18.2	0.001
	Time to task failure (s)	Sham	–	64.7 ± 15.9	

**Table 5:** Effects of tDCS on motor and neuromuscular outcomes (M1-targeted studies).

Study	Variable	Group	Before	After	*p*-Value
Moshashaei et al. [11]	Vastus Lateralis	Active tDCS	95.82 ± 36.29	141.70 ± 8.74	ns
	Vastus Lateralis	Sham	117.02 ± 33.53	129.47 ± 29.99	
	Vastus Medialis	Active tDCS	89.89 ± 37.43	122.18 ± 41.63	0.18
	Vastus Medialis	Sham	106.97 ± 39.36	118.06 ± 43.23	
	Gluteus Maximus	Active tDCS	60.96 ± 23.61	83.34 ± 27.04	0.014
	Gluteus Maximus	Sham	70.01 ± 20.13	34.80 ± 12.98	
	Gluteus Medius	Active tDCS	63.32 ± 20.06	80.22 ± 21.47	0.04
	Gluteus Medius	Sham	50.50 ± 15.35	62.02 ± 15.21	
	Semitendinosus	Active tDCS	41.97 ± 18.51	73.39 ± 32.21	0.01
	Semitendinosus	Sham	40.32 ± 11.23	51.21 ± 13.94	
	Biceps Femoris	Active tDCS	41.52 ± 21.71	62.86 ± 24.68	0.009
	Biceps Femoris	Sham	59.75 ± 33.29	70.34 ± 29.40	
	Gastrocnemius	Active tDCS	58.87 ± 24.30	93.01 ± 26.49	0.0001
	Gastrocnemius	Sham	66.90 ± 30.41	86.63 ± 39.10	
Mesquita et al. [12]	CMJ	Active tDCS	35.7	35.5	0.560
	CMJ	Sham	35.9	35.0	
	Total number of kicks	Active tDCS	93.0	93.8	0.685
	Total number of kicks	Sham	94.7	95.0	
	Kick decrement index (%)	Active tDCS	8.4	8.3	0.667
	Kick decrement index (%)	Sham	8.3	8.5	
Mesquita et al. [17]	Time to exhaustion	Active tDCS	14.6 ± 1.9	–	0.74
	Time to exhaustion	Sham	14.9 ± 2.1	–	
	Peak kicking frequency	Active tDCS	52.0 ± 6.5	–	0.33
	Peak kicking frequency	Sham	53.6 ± 7.0	–	
Moshashaei et al. [15]	Vastus Lateralis (SLS)	Active tDCS	27.35 ± 60.17	43.72 ± 110.77	0.001
	Vastus Lateralis (SLS)	Sham	20.87 ± 56.22	25.03 ± 68.08	
	Vastus Medialis (SLS)	Active tDCS	28.03 ± 61.28	41.28 ± 102.96	0.50
	Vastus Medialis (SLS)	Sham	26.12 ± 70.82	26.87 ± 76.58	
	Gluteus Maximus (SLS)	Active tDCS	10.95 ± 21.53	19.87 ± 24.07	0.52
	Gluteus Maximus (SLS)	Sham	10.32 ± 26.42	12.93 ± 20.57	
Kamali et al. [10]	Reaction time	Active tDCS	0.51 ± 0.18	–	0.0001
	Reaction time	Sham	0.55 ± 0.17	–	
	Selective attention	Active tDCS	3.4 ± 5.2	–	0.0001
	Selective attention	Sham	6.47 ± 6.1	–	
	Visual-spatial working memory	Active tDCS	4.73 ± 1.4	–	0.17
	Visual-spatial working memory	Sham	4.4 ± 1.5	–	
	Endurance time	Active tDCS	42.2 ± 27.5	–	0.55
	Endurance time	Sham	40.6 ± 35.1	–	
	Number of kicks during endurance	Active tDCS	89.4 ± 79.4	–	0.06
	Number of kicks during endurance	Sham	98.4 ± 63.6	–	
Kamali et al. [19]	Reaction time of right hands	Real-tDCS	300 ms		0.0001
	Reaction time of right hands	Sham-tDCS	330 ms		
	Reaction time of left hands	Real-tDCS	325 ms		0.0006
	Reaction time of left hands	Sham-tDCS	360 ms		
	Selective attention assessment	Real-tDCS	50		0.0003
	Selective attention assessment	Sham-tDCS	95		
	Visuospatial memory assessment	Real-tDCS	6.0		0.7202
	Visuospatial memory assessment	Sham-tDCS	5.8		
	Spatial span task	Real-tDCS	6.7		0.6576
	Spatial span task	Sham-tDCS	6.5		
	Double trouble task	Real-tDCS	80		0.0719
	Double trouble task	Sham-tDCS	75		
	Hand Grip right hand	Real-tDCS	52		0.9806
	Hand Grip right hand	Sham-tDCS	52		
	Hand Grip left hand	Real-tDCS	50		0.9776
	Hand Grip left hand	Sham-tDCS	50		

**Table 6:** The certainty of evidence for the main outcome domains was qualitatively assessed using the GRADE.

Outcome	Studies	Risk of Bias	Inconsistency	Indirectness	Imprecision	Certainty
Reaction time	3 (Kamali et al. [10,19])	Serious	Serious	Moderate	Serious	Low
Attention	3 (Kamali et al. [10,19]; Selitrenikova et al. [14])	Serious	Moderate	Moderate	Serious	Low
Memory	4 (Kamali et al. [10,19]; Selitrenikova et al. [14])	Serious	Serious	Serious	Serious	Very low
Neuromuscular activation	2 (Moshashaei et al. [11]; Moshashaei et al. [15])	Moderate	Moderate	Moderate	Serious	Low
Anxiety	1 (Seyfaddini Zarandi et al. [16])	Serious	NA	Moderate	Serious	Very low
Endurance	3 (Kamali et al. [10]; Mesquita et al. [12,17])	Moderate	Moderate	Low	Serious	Low

## Data Availability

No new data were created or analyzed in this study. Data sharing is not applicable to this article.

## Supplementary Materials

The following supporting information can be downloaded at: https://www.mdpi.com/article/10.3390/brainsci16101052/s1, Table S1: PRISMA 2020 checklist.
